# Folic acid in pregnancy and mortality from cancer and cardiovascular disease: further follow-up of the Aberdeen folic acid supplementation trial

**DOI:** 10.1136/jech-2014-205324

**Published:** 2015-04-08

**Authors:** Caroline M Taylor, Charlotte Atkinson, Chris Penfold, Sohinee Bhattacharya, Doris Campbell, George Davey Smith, Sam Leary, Andy Ness

**Affiliations:** 1 NIHR Biomedical Research Unit in Nutrition, Diet and Lifestyle, University of Bristol, Bristol, UK; 2 Dugald Baird Centre for Research on Women's Health, University of Aberdeen, Aberdeen Maternity Hospital, Aberdeen, UK; 3 Institute of Applied Health Sciences, University of Aberdeen, Aberdeen, UK; 4 MRC Integrative Epidemiology Unit, School of Social and Community Medicine, University of Bristol, Bristol, UK

**Keywords:** CANCER, CANCER: BREAST, MORTALITY, EPIDEMIOLOGY, MATERNAL HEALTH

## Abstract

**Background:**

Supplemental periconceptional folic acid is recommended to reduce the risk of fetal neural tube defects. A previous report indicated an elevated risk of breast cancer and all cancer deaths in later life among women randomised by alternate allocation to high-dose (5 mg/day) folic acid in pregnancy compared with placebo; however, findings were based on small numbers of cases. Our aim was to extend the previous analysis by including data from an additional 10 years of follow-up.

**Methods:**

Records of participants in a large (n=2928) trial of folate supplementation (5 or 0.2 mg folic acid, or placebo) in pregnancy in the 1960s were linked to central registries in Scotland. Unadjusted and adjusted HRs were calculated for all-cause, cardiovascular, all cancer and breast cancer mortality, and all cancer and breast cancer morbidity. Analyses were done using (1) data from the time of the previous linkage (2002) to March 2013; and (2) data from 1980 to March 2013.

**Results:**

There was no evidence to suggest an excess risk of morbidity or mortality in either supplementation group compared with placebo for 2002–2013 and no associations were seen for the full time period (1980–2013).

**Conclusions:**

Findings from this extended follow-up do not support our previous observation of an elevated risk of mortality from breast cancer or all cancers in later life among women who had taken 5 mg folic acid/day during pregnancy. Furthermore, there were no associations with risk of mortality from all-causes, all cancers or cardiovascular disease.

## Introduction

Folate (found in foods) or folic acid (synthetic form) acts as a coenzyme in several single-carbon transfers. These transfers lead to the synthesis of purine nucleotide and deoxythymidylic acid which, in turn, are essential for DNA and RNA synthesis. It is well established that periconceptional folic acid supplementation substantially reduces the risk of neural tube defects in offspring.^1–5^ Several countries mandate the fortification of food products with folic acid (USA, Canada, etc^6^), whereas others such as the UK advocate periconceptual supplementation (0.4 mg/day) until 12 weeks of gestation.^7^

The synthetic form of folic acid is nearly twice as bioavailable as folate occurring naturally in food and is more stable. Supplementation therefore leads to relatively higher blood levels of the unmetabolised form that remains in the blood for longer than the natural form.^8^ The health consequences of this are not completely understood and there are concerns that fortification and supplementation could cause adverse effects, including masking of vitamin B_12_ deficiency, neurotoxicity, drug interactions, reduced zinc absorption and hypersensitivity reactions.^9^

It has also been suggested that there is an association between increased folic acid intake and cancer promotion,^9^ but findings have been inconsistent. Evidence from epidemiological studies and clinical trials suggests that there is an inverse relationship between folate intake and the incidence of colorectal cancer,^10–12^ but a recent meta-analysis did not find any evidence of an effect of 5 years of supplementation on overall and site-specific cancer incidence.^13^ Some animal studies have shown that supplementation increases the risk of experimental carcinogenesis^14^ and an ecological study suggested that mandatory folic acid fortification in the mid-1990s in the USA and Canada led to an increase in rates of colorectal cancer.^15^ In a recent follow-up of the aspirin/folate Polyp Prevention Trial, in which folic acid was trialled as a chemotherapeutic agent, there was an increased risk of prostate cancer in men who had been randomised to supplemental folic acid compared with the placebo group.^16^ Evidence on the association of folate supplementation with the incidence of breast cancer is equally difficult to interpret, and is further complicated by studies reporting dietary folate intake alone rather than total folate intake.^17^ A meta-analysis showed an inverse association between dietary folate and risk of breast cancer in case–control studies, but not prospective studies, with a potential role for folate in attenuating the increased risk of breast cancer associated with alcohol consumption.^18^ The timing and dose of folic acid may be critical in determining either beneficial or adverse effects on cancer risk and may in part explain the conflicting results from both epidemiological studies and clinical trials. The inconsistency in findings could also potentially be due to a ‘dual effect’ of folate: it has recently been suggested that folate supplementation in people with (undetected) precancerous cells may increase the rate of malignant transformation of such cells,^19^ possibly by causing aberrant DNA methylation,^20^ but in those without precancerous cells folate it may confer a protective effect.

Folic acid supplementation may, however, be protective against cardiovascular disease (CVD): high levels of homocysteine have been associated with an increased risk of CVD, and folic acid supplementation is known to reduce homocysteine levels.^21^ However, in meta-analyses of randomised controlled trials, folic acid supplementation was not shown to reduce the risk of CVD among individuals with or without a prior history of vascular disease or other predisposing factors.^22–24^ In trials that reported the risk stratified by baseline homocysteine level, the risk of CVD was increased in participants with higher homocysteine levels and decreased in those with lower levels, suggesting that folic acid may influence the progression of atherosclerotic disease through pathways that are not dependent on the lowering of homocysteine levels.^23^

The long-term effects of folic acid supplementation during pregnancy on maternal mortality and morbidity in later life have not been well studied. In a previous follow-up of a large (n=2928) trial of folic acid supplementation in pregnancy from the 1960s it was shown that randomisation to a high dose of folic acid (5 mg/day, by alternate allocation) was associated with a higher risk of mortality from cancer (HR=1.70, 95% CI 1.06 to 2.72) and possibly breast cancer specifically (HR=2.02, 95% CI 0.88 to 4.72) in later life.^25^ However, the analyses were based on relatively small numbers of cases (112 deaths due to cancer and 31 to breast cancer), and the findings may have been due to chance. The aim of the present study was to extend the previous analysis by ascertaining the vital status and cause of death up to March 2013, thereby adding about an additional 10 years of follow-up. The primary outcomes were all-cause, all cancer, breast cancer and cardiovascular mortality; the secondary outcomes were all cancer and breast cancer morbidity.

## Materials and methods

### Participants, intervention and compliance

The parent study has been described in detail elsewhere.^26–28^ Briefly, from June 1966 to June 1967, 3187 potentially eligible women (women booking for antenatal care at <30 weeks gestation who were resident in Aberdeen, UK) were invited to enter a double-blind randomised controlled trial to examine the effects of folic acid supplementation on pregnancy outcomes. In all, 2928 women were randomised by alternate allocation to receive either 0.2 mg folic acid/day (n=466, 15.6%), 5 mg folic acid/day (n=485, 16.6%) or a placebo (n=1977, 67.5%). Compliance was assessed by self-report and by measurement of folate status. In the placebo group, 1.9% reported that they had not taken their tablets regularly, compared with 1.7% in the group taking 0.2 mg folic acid and 3.2% in the group taking 5 mg. Prior to randomisation, serum folate concentrations were similar in the three groups; a dose–response relationship was seen after randomisation.^28^

### Record linkage: morbidity and mortality data

For determination of the primary outcomes (all-cause, all cancer, breast cancer and cardiovascular mortality), records of the participants in this trial were linked to those held by the Scottish NHS Central Registry in Edinburgh to provide data on vital status and cause of death for those who had died from January 1980 to March 2013. As per the previous follow-up, the cause of death was assigned using the following codes: cardiovascular mortality International Classification of Diseases, Ninth Revision (ICD-9) 410–414, 430–439 and ICD-10 120–125, 160–169; all cancers ICD-9 140–208, ICD-10 C00–C97; breast cancer ICD-9–174, ICD-10–C50). For determination of the secondary outcomes (all-cancer and breast cancer morbidity), the records of participants were also linked to the Scottish Morbidity Records (SMR), specifically to the hospital admissions (SMR01) and cancer registrations (SMR06), using both deterministic matching on the Community Health Index (CHI) numbers and probabilistic matching on names, dates of birth and postcodes of residence. The SMR registers are held by the Information and Services Division (ISD) of National Health Service (NHS) Scotland.

### Data analysis

Baseline data were summarised as means and SD (continuous data) or number and percentage (categorical data). We calculated unadjusted and adjusted HRs for the primary and secondary outcomes using Stata v.13.1 (StataCorp, College Station, Texas, USA). In contrast to our previous data linkage we do not have data on mortality prior to 1980 as the costs of obtaining such data were prohibitive. We are therefore unable to completely replicate the findings that were presented in Charles *et al*.^25^ In the present study, analyses for mortality and morbidity were carried out for two time periods: 2002–2013 (to see if the findings reported previously were also seen in the ‘new’ time period) and for the entire time period for which data in the current linkage are available (ie, 1980–2013; the mean age of the participants was approximately 73 years at the end of follow-up). As per the previous analysis,^25^ we adjusted for maternal age, smoking, height, weight, social class, parity and gestational age. However, unlike the previous analysis, we were unable to adjust for systolic blood pressure because we did not have access to data on the full cohort.

## Results

The baseline characteristics of the women in the three treatment groups were broadly comparable (table 1), with the exception of a greater percentage of women having ≥4 pregnancies in the placebo group, as discussed previously.^28^ For the ‘new’ time period (2002–2013), there was no evidence to suggest an excess risk of all-cause mortality, CVD mortality or breast cancer mortality in either supplementation group (0.2 or 5 mg/day) compared with placebo (table 2). There was a suggestion of a lower risk of all cancer deaths in unadjusted (HR=0.36, 95% CI 0.16 to 0.83, p=0.02) and adjusted analyses (HR=0.43, 95% CI 0.19 to 1.00, p=0.05) for 5 mg folic acid/day compared with placebo but not for 0.2 mg/day, but this finding was based on only six deaths in the 5 mg/day group. No associations were observed when looking at the full time period (1980–2013; table 3). When the two supplementation groups were combined there was no evidence of an association between folic acid supplementation and mortality from all-causes, CVD, all cancers or breast cancer (tables 2 and 3). There were no associations between folic acid supplementation (at either dose) and morbidity from all cancers or breast cancer (all associations p>0.05; tables 4 and 5).

**Table 1:** Baseline characteristics of the women enrolled in the Aberdeen folic acid supplementation trial 1966–1967 (n=2928)

	Placebo (n=1977)	Folic acid supplement (mg/day)	
		0.2 (n=466)	5 (n=485)*
Age (years)*	26.0 (5.6)	25.8 (5.5)	25.5 (5.2)
Gestational age at booking (weeks)	17.5 (5.3)	16.9 (4.8)	17.7 (5.6)
Weight at booking (kg)†	59.6 (9.5)	59.0 (8.9)	59.6 (9.1)
Height (cm)‡	159.5 (5.9)	159.4 (6.4)	159.5 (6.1)
Smoked at booking, n (%)§	840 (45.8)	188 (42.2)	203 (44.7)
Social class code, n (%)¶			
I	122 (6.2)	31 (6.7)	34 (7.0)
II	219 (11.1)	44 (9.4)	49 (10.1)
IIIN	204 (10.3)	61 (13.1)	63 (13.0)
IIIM	768 (38.8)	170 (36.5)	146 (29.9)
IV	330 (16.7)	70 (15.0)	91 (18.8)
V	164 (8.3)	43 (9.2)	44 (9.1)
Undefined or armed forces	170 (8.6)	47 (10.1)	59 (12.2)
Parity, n (%)			
0	734 (37.1)	198 (42.5)	196 (40.4)
1–3	904 (45.7)	216 (46.4)	224 (46.2)
≥4	339 (17.1)	52 (11.2)	65 (13.4)

Values are mean±SD.

Adapted with copyright permission from Charles *et al*.^28^

*Age available for 2868 (98%) women.

†Weight at booking available for 2813 (96.1%) women.

‡Height available for 2860 (97.7%) women.

§Smoking status at booking available for 2734 (93.4%) women.

¶Based on the husband's or partner's occupation at the time of delivery.

**Table 2:** Unadjusted and adjusted* HRs for mortality between 2002 and 2013 from all-causes, cardiovascular disease, cancer and breast cancer in the groups given folic acid supplements in the Aberdeen folic acid supplementation trial, 1966–1967

	n	Unadjusted HR (95% CI; p value)	p For trend	Adjusted HR* (95% CI; p value)	p For trend
*Mortality risk in the two separate supplement groups*					
All-cause mortality					
Placebo	163	1.00	0.46	1.00	0.89
0.2 mg folic acid	45	1.21 (0.86 to 1.67; 0.28)		1.27 (0.90 to 1.81; 0.17)	
5 mg folic acid	33	0.79 (0.54 to 1.16; 0.23)		0.87 (0.58 to 1.31; 0.52)	
Cardiovascular mortality					
Placebo	37	1.00	0.18	1.00	0.17
0.2 mg folic acid	7	0.83 (0.37 to 1.86; 0.65)		1.00 (0.44 to 2.27; 1.00)	
5 mg folic acid	15	1.63 (0.89 to 2.97; 0.11)		1.67 (0.86 to 3.26; 0.13)	
All cancer deaths					
Placebo	68	1.00	0.06	1.00	0.19
0.2 mg folic acid	20	1.27 (0.77 to 2.09; 0.35)		1.42 (0.84 to 2.39; 0.19)	
5 mg folic acid	6	0.36 (0.16 to 0.83; 0.02)		0.43 (0.19 to 1.00; 0.05)	
Breast cancer mortality					
Placebo	16	1.00	0.28	1.00	0.31
0.2 mg folic acid	2	0.54 (0.12 to 2.34; 0.41)		0.52 (0.12 to 2.31; 0.39)	
5 mg folic acid	2	0.51 (0.12 to 2.23; 0.37)		0.54 (0.12 to 2.41; 0.42)	
*Mortality risk in the two supplement groups combined*					
All-cause mortality					
Placebo	163	1.00		1.00	
Supplemented	78	0.99 (0.75 to 1.30; 0.93)		1.07 (0.80 to 1.43; 0.64)	
Cardiovascular mortality					
Placebo	37	1.00		1.00	
Supplemented	22	1.25 (0.74 to 2.11; 0.41)		1.33 (0.75 to 2.35; 0.33)	
All cancer deaths					
Placebo	68	1.00		1.00	
Supplemented	26	0.80 (0.51 to 1.26; 0.34)		0.91 (0.57 to 1.46; 0.70)	
Breast cancer mortality					
Placebo	16	1.00		1.00	
Supplemented	4	0.52 (0.18 to 1.57; 0.25)		0.53 (0.17 to 1.62; 0.27)	

*Adjusted for maternal age, smoking, height, weight, social class, parity and gestational age.

**Table 3:** Unadjusted and adjusted* HR’s for mortality between 1980 and 2013 from all-causes, cardiovascular disease, cancer, and breast cancer in the groups given folic acid supplements in the Aberdeen folic acid supplementation trial, 1966–1967

	n	Unadjusted HR (95% CI; p value)	p For trend	Adjusted HR* (95% CI; p value)	p For trend
*Mortality risk in the two separate supplement groups*					
All-cause mortality					
Placebo	289	1.00	0.88	1.00	0.22
0.2 mg folic acid	78	1.16 (0.91 to 1.49; 0.24)		1.23 (0.95 to 1.81; 0.12)	
5 mg folic acid	70	0.97 (0.75 to 1.26; 0.82)		1.13 (0.86 to 1.48; 0.39)	
Cardiovascular mortality					
Placebo	69	1.00	0.34	1.00	0.22
0.2 mg folic acid	13	0.82 (0.45 to 1.48; 0.50)		0.92 (0.51 to 1.68; 0.80)	
5 mg folic acid	23	1.35 (0.84 to 17; 0.21)		1.45 (0.87 to 2.41; 0.15)	
All cancer deaths					
Placebo	132	1.00	0.62	1.00	0.69
0.2 mg folic acid	36	1.17 (0.81 to 1.69; 0.41)		1.30 (0.89 to 1.90; 0.18)	
5 mg folic acid	27	0.84 (0.55 to 1.27; 0.40)		1.00 (0.65 to 1.52; 0.99)	
Breast cancer mortality					
Placebo	36	1.00	0.77	1.00	0.61
0.2 mg folic acid	8	0.95 (0.44 to 2.04; 0.90)		0.98 (0.45 to 2.13; 0.96)	
5 mg folic acid	10	1.14 (0.56 to 2.29; 0.72)		1.24 (0.61 to 2.51; 0.56)	
*Mortality risk in the two supplement groups combined*					
All-cause mortality					
Placebo	289	1.00		1.00	
Supplemented	148	1.06 (0.87 to 1.29; 0.60)		1.17 (0.95 to 1.44; 0.14)	
Cardiovascular mortality					
Placebo	69	1.00		1.00	
Supplemented	36	1.09 (0.73 to 1.63; 0.67)		1.18 (0.77 to 1.80; 0.45)	
All cancer deaths					
Placebo	132	1.00		1.00	
Supplemented	63	0.98 (0.73 to 1.33; 0.91)		1.13 (0.82 to 1.54; 0.45)	
Breast cancer mortality					
Placebo	36	1.00		1.00	
Supplemented	18	0.99 (0.55 to 1.76; 0.97)		1.05 (0.58 to 1.89; 0.87)	

*Adjusted for maternal age, smoking, height, weight, social class, parity and gestational age.

**Table 4:** Unadjusted and adjusted* HRs for morbidity between 2002 and 2013 from all cancer and breast cancer in the groups given folic acid supplements in the Aberdeen folic acid supplementation trial, 1966–1967

	n	Unadjusted HR (95% CI; p value)	p For trend	Adjusted HR (95% CI; p value)*	p For trend
All cancer morbidity					
Placebo	134	1.00	0.46	1.00	0.88
0.2 mg folic acid	34	1.06 (0.73 to 1.54; 0.76)		1.18 (0.80 to 1.75; 0.41)	
5 mg folic acid	27	0.82 (0.54 to 1.24; 0.35)		0.90 (0.59 to 1.39; 0.65)	
Breast cancer morbidity					
Placebo	34	1.00	0.77	1.00	0.98
0.2 mg folic acid	6	0.74 (0.31 to 1.76; 0.50)		0.86 (0.35 to 2.08; 0.74)	
5 mg folic acid	8	0.96 (0.45 to 2.08; 0.93)		1.06 (0.46 to 2.44; 0.89)	

*Adjusted for maternal age, smoking, height, weight, social class, parity and gestational age.

**Table 5:** Unadjusted and adjusted* HRs for morbidity between 1980 and 2013 from all cancer and breast cancer in the groups given folic acid supplements in the Aberdeen folic acid supplementation trial, 1966–1967

	n	Unadjusted HR (95% CI; p value)	p For trend	Adjusted HR* (95% CI; p value)	p For trend
All cancer morbidity					
Placebo	292	1.00	0.97	1.00	0.88
0.2 mg folic acid	68	0.98 (0.75 to 1.28; 0.88)		1.06 (0.81 to 1.39; 0.68)	
5 mg folic acid	72	1.01 (0.78 to 1.31; 0.93)		1.12 (0.86 to 1.46; 0.40)	
Breast cancer morbidity					
Placebo	98	1.00	0.67	1.00	0.38
0.2 mg folic acid	19	0.82 (0.50 to 1.34; 0.42)		0.86 (0.52 to 1.42; 0.57)	
5 mg folic acid	28	1.17 (0.77 to 1.78; 0.47)		1.29 (0.84 to 2.00; 0.25)	

*Adjusted for maternal age, smoking, height, weight, social class and gestational age.

## Discussion

In this extended follow-up of women who had participated in the Aberdeen folate trial in the 1960s, we did not see any strong evidence of an association between either dose of folic acid taken during pregnancy and mortality from cancer or CVD in later life.

In the previous analysis,^25^ there was a suggestion of an elevated risk of mortality from all cancers, and possibly from breast cancer specifically, among women who had received 5 mg folic acid/day during pregnancy. For the present study, we conducted separate analyses using only the newly acquired data (to see if the findings reported previously were also seen in this ‘new’ time period), and using all data for the available time period (which included data from the previous analysis plus the additional years of follow-up). In contrast to our previous findings, in the ‘new’ time period there was a suggestion of a decreased risk of all cancers among women in the high dose folic acid group (5 mg/day). However, this finding was based on a very small number of cases (n=6) and no associations were seen for the full time period. Taken together, this suggests that the previous findings in regards to an adverse effect on mortality from all cancers and possibly breast cancer, and the current suggestion of a potential protective effect on mortality from all cancers, may be due to chance. Similar to our previous analysis, we saw no effect of either dose of folic acid during pregnancy on all-cause mortality or cardiovascular mortality in later life when looking at either the ‘new’ time period alone or all data that were available. There was no effect on all-cancer or breast cancer morbidity in later life.

A ‘dual effect’ theory for the effects of folic acid has previously been proposed by Kim^19^ whereby folic acid in people with undetected lesions at the time of supplementation may promote the growth of such lesions, but there may be no (or protective) effects in those without lesions. This could perhaps explain the contrasting effects seen at differing time periods in our studies; however, the length of time of supplementation in the present study may be too short for this theory to be relevant. In addition, as noted earlier, the number of cases in the ‘new’ time period was too small to draw any meaningful conclusions.

Pregnancy is likely to be to be a critical time for the determination of risk of subsequent breast cancer. To the best of our knowledge, no other studies have undertaken a long-term follow-up after folic acid supplementation in pregnancy, a time during which breast tissue undergoes anatomical changes in response to hormonal action and develops the ability to secrete milk.^29^ Studies in animal models have provided consistent evidence that the hormonal milieu of pregnancy is protective against mammary tumours.^30^ In humans, however, the associations are more complex. Childbirth is followed by a short period of increased risk,^31^ but early age at completion of first pregnancy, however, seems to be protective.^32^ The mechanisms are poorly understood, but may involve the marked changes in endogenous hormone concentrations, particularly oestrogen.^33^ ^34^ Several studies have found dietary folate to be protective against breast cancer,^35–37^ but it is not known whether supplementary folic acid in pregnancy modifies this association.

Meta-analyses have provided conflicting information on the role of long-term folic acid supplementation or dietary intakes in non-pregnant participants on overall cancer incidence and site-specific cancers. A recent meta-analysis of randomised trials found no effect of 5 years of supplementation on the overall cancer incidence or incidence of site-specific cancers (including large intestine, prostate, lung and breast).^13^ With regard to dietary intakes, a meta-analysis on folate intake reported an association of higher intakes with a decreased risk of colorectal cancer of 8–15% depending on the type of study.^10^ Another meta-analysis reported a decreased risk of breast cancer with increasing dietary folate intake (although no association was seen with blood folate concentration).^17^ It is likely that the role of folate in cancer is complex, and there may be differing effects of natural (dietary) folate versus supplements. With regard to CVD, meta-analyses of randomised controlled trials also showed no effect of folic acid supplementation on the risk of CVD in participants with or without a history of vascular disease.^23^ ^24^ Although the studies included in these meta-analyses involved longer periods of supplementation (up to 7 years) than the present study, the length of follow-up has generally been much shorter (<7 years).

Our study has a number of strengths. The parent study was a randomised controlled trial that included random allocation (treatments were sequentially allocated but effectively random^38^), adequate concealment and subjective and objective evidence of good compliance, and the supplementation groups were similar at baseline. In addition, to the best of our knowledge this is the only long-term follow-up of women who had taken two different doses of folic acid supplements during pregnancy. There are some limitations to this study. Despite an additional 10 years of follow-up since our last data linkage, the number of deaths was still relatively small, which resulted in wide CIs around some of the hazard estimates. Exclusion of deaths before 1980 is likely to have resulted in a small underestimation of mortality and morbidity, as would being confining the linkage to those participants still resident in Scotland. The dose and timing of folate supplementation in the trial was different from current UK recommendations of 0.4 mg/day periconceptually,^7^ which may limit the generalisability of the findings to the present day population. Furthermore, the results may not be generalisable to a wider UK population or to populations in other countries; this limitation may be amplified by possible heterogeneity in the *MTHFR* gene, which may be related to the risk of colorectal and other gastrointestinal cancers.^39^ ^40^

## Conclusion

We did not find any strong evidence to suggest an effect of either dose of folic acid taken during pregnancy on mortality from all-causes, cancer or CVD in later life, or on morbidity from all cancer or breast cancer. Further data linkage studies could be conducted in the future to confirm whether this remains the case with a longer period of follow-up and additional deaths.

**What is already known on this subject?**

- The long-term effects of folic acid supplementation during pregnancy on maternal mortality in later life have not been well studied. In a previous follow-up of a large trial of folic acid supplementation in pregnancy from the 1960s it was shown that randomisation to a high dose of folic acid (5 mg/day) was associated with a higher risk of mortality from cancer and possibly breast cancer specifically. However, the analyses were based on relatively small numbers of cases and the findings may have been due to chance.

**What this study adds?**

- We did not find any strong evidence to suggest an effect of either dose of folic acid taken during pregnancy on mortality from all-causes, cancer or cardiovascular disease in later life with an extended follow-up time. Further data linkage studies should be conducted in the future to confirm whether this remains the case with a longer period of follow-up and additional deaths.
